# A Study on the Effect of Copper Methionine in Alleviating Yellowing in Channel Catfish

**DOI:** 10.3390/vetsci13070683

**Published:** 2026-07-14

**Authors:** Dongyu Huang, Lu Zhang, Chunyu Xue, Leimin Zhang, Mingchun Ren, Xiaodi Xu, Hualiang Liang, Haifeng Mi

**Affiliations:** 1 Key Laboratory of Integrated Rice-Fish Farming Ecology, Ministry of Agriculture and Rural Affairs, Freshwater Fisheries Research Center, Chinese Academy of Fishery Sciences, Wuxi 214081, China; huangdongyu@ffrc.cn (D.H.); renmc@ffrc.cn (M.R.); xuxiaodi@ffrc.cn (X.X.); 2 Tongwei Agricultural Development Co., Ltd., Key Laboratory of Aquatic Nutrition and Smart Farming, Ministry of Agriculture and Rural Affairs, Aquatic Health and Intelligent Aquaculture Key Laboratory of Sichuan Province, Chengdu 610093, China; zhangl21@tongwei.com; 3 Wuxi Fisheries College, Nanjing Agricultural University, Wuxi 214081, China; 2022213004@stu.njau.edu.cn (C.X.); 2022113002@stu.njau.edu.cn (L.Z.)

**Keywords:** copper methionine, pigmentation, antioxidant capacity, channel catfish

## Abstract

An 8-week trial evaluated dietary copper methionine (CM) on body and flesh color in yellowed channel catfish. Basic diets were supplemented with CM at 0 (control, Cu1), 7 (Cu2), 14 (Cu3), 21 (Cu4), and 27 (Cu5) mg/kg, with corresponding measured copper levels of 10.82, 12.63, 14.35, 15.58, and 17.40 mg/kg. In skin, GSH levels in Cu2-Cu5 groups and GSH-Px activities in Cu2-Cu4 groups were increased, and in muscle, CAT activities in Cu3-Cu5 groups and GSH levels in Cu4-Cu5 groups were increased. Gene expression analysis showed downregulated *keap1* (Cu2–Cu4 in skin; Cu3–Cu5 in muscle) and *nfκb* (Cu2–Cu5 in skin), upregulated *nrf2* (Cu3–Cu5 in skin; Cu4 in muscle) and *il-10* (Cu4 in skin). Colorimetric analysis showed reduced the values of b* in skin (Cu3–Cu5) and muscle (Cu2–Cu5). Uranidin content decreased in skin (Cu3–Cu5) and muscle (Cu4–Cu5), while melanin and tyrosinase increased in skin (melanin: Cu2–Cu3; tyrosinase: Cu3–Cu4) and muscle (Cu3–Cu5). The expression of melanogenesis-related genes (*mitf*, *tyr*, *etb*, *camk2*, *slc24a5*) was increased in skin and muscle of Cu3-Cu5 groups, while the uranidin synthesis gene *xdh* was downregulated. Overall, optimal effects were observed at 14–21 mg/kg CM (measured copper: 14.35–15.58 mg/kg).

## Simple Summary

This study focuses on the role of copper methionine in restoring body color in yellowed channel catfish. By adding different concentrations of copper methionine to the feed, the study investigates its effects on body color parameters, melanin and uranidin content, tyrosinase activity, and antioxidant-related indicators in yellowed channel catfish, with the aim of determining the optimal addition level of copper methionine for alleviating yellowing and elucidating its potential mechanisms of action. The aim is to provide a scientific basis for the development of safe and effective feed additives for body color regulation, to address the challenge of yellowing in channel catfish farming, and to refine the theoretical framework of trace element nutrition and body color regulation in fish, thereby contributing to the green and healthy development of the industry.

## 1. Introduction

Copper is one of the most abundant metallic elements in living organisms and is also one of the essential trace elements required for optimal growth performance and health in aquatic animals [1]. As a cofactor for redox enzymes such as cytochrome C oxidase, monoamine oxidase and copper-zinc superoxide dismutase (Cu/Zn-SOD), it participates in the formation of copper chelates, antioxidant enzymes, cytochrome oxidases and various proteins associated with metabolic functions, playing a vital role in numerous biological processes such as redox reactions, antioxidant defense and neuropeptide synthesis [2,3]. Furthermore, the antifungal properties of copper have been extensively studied and hold great promise for application in the aquaculture sector [4,5].

Fish body color is a well-recognized visual indicator of nutritional and physiological status, reflecting the overall health condition of the animal. Its formation and regulation involve complex mechanisms, primarily relying on the synergistic action of pigment cells—such as melanocytes and xanthophores—as well as the synthesis and deposition of pigments such as melanin and carotenoids [6]. Copper plays an indispensable role in the regulation of fish body color. As a key component of tyrosinase, copper is directly involved in the biosynthesis of melanin; insufficient copper supply leads to reduced tyrosinase activity and impaired melanin synthesis, thereby causing abnormal phenomena such as lightening, yellowing or albinism in fish [7]; simultaneously, copper maintains body color stability by regulating the function of the antioxidant system and reducing free radical damage to pigment cells [8]. Conversely, an imbalance in copper supply disrupts the body’s metabolic equilibrium, leading to abnormal body color through the inhibition of melanin synthesis, whilst also exacerbating oxidative stress and worsening the symptoms of color abnormalities. Therefore, the addition of copper to feed is a crucial approach for regulating fish body color. Copper preparations traditionally used in aquaculture are predominantly inorganic copper compounds (such as copper sulphate and copper chloride). Whilst these copper sources are widely available and relatively inexpensive, they suffer from low bioavailability and a tendency to form insoluble complexes with other substances within the fish, leading to accumulation. This not only makes it difficult to precisely meet the nutritional copper requirements of fish but also increases the risk of supply imbalances due to improper dosage control, thereby exacerbating oxidative stress and the risk of abnormal body color [9,10]. Consequently, identifying new copper sources or developing copper preparations with high bioavailability is a key strategy for promoting more efficient copper utilization in fish.

Compared with inorganic copper, organic copper chelates formed with amino acids, peptides or other organic compounds offer high bioavailability and absorption rates [11]. Methionine copper refers to an organometallic chelate formed by the coordination of copper ions (Cu^2+^) with methionine; its molecular structure typically entails the binding of copper ions to one or two methionine molecules in a 1:1 or 1:2 molar ratio, forming a stable five- or six-membered ring structure [12]. While there has been considerable research on the application of CM in aquaculture, much of it has focused on promoting growth and improving feed conversion efficiency [13,14,15]; research into its ability to improve abnormal body coloration in fish by regulating pigment synthesis pathways and alleviating oxidative stress has not yet been thoroughly explored. In particular, systematic studies on its targeted application in alleviating body yellowing remain lacking.

The channel catfish, also known as *Ictalurus punctatus*, is native to North America and was introduced to China by the Hubei Provincial Fisheries Science Institute in 1984. This species possesses excellent aquaculture characteristics, including rapid growth rate, broad omnivorous diet, and strong adaptability. Moreover, its meat is tender, delicious, free of intermuscular spines, and nutritionally balanced. Since its introduction, it has swiftly become a favored freshwater fish among consumers and has now developed into one of the core species in China’s freshwater aquaculture industry. With the promotion of aquaculture techniques and the industrial scaling-up, the scale and output of channel catfish farming in China have continued to rise. In 2024, the national output of channel catfish reached 526,249 tons, indicating a strong momentum in industrial development. However, under intensive artificial farming conditions, the phenomenon of skin and muscle yellowing in channel catfish occurs frequently. Yellowed individuals are widely regarded as unqualified products in the market, resulting in a lower market price compared to those with normal body color. This significantly impacts the economic benefits of farmers and restricts the high-quality and sustainable development of the channel catfish farming industry.

Therefore, given the important role of copper in regulating fish body color and the higher bioavailability and safety of CM, the aim of this study is to investigate the effects of adding different concentrations of CM to the feed on the body color parameters, melanin and xanthophyll content, tyrosinase activity and antioxidant-related indicators in yellow-channel catfish, with a view to determining the optimal addition level of CM for alleviating yellowing and identifying its potential mechanisms of action.

## 2. Materials and Methods

### 2.1. Experimental Diets

In this experiment, commercial diet served as the control (Table 1). Five experimental diets were formulated by supplementing the control diet with copper methionine (CM) at levels of 0 mg/kg (Cu1), 7 mg/kg (Cu2), 14 mg/kg (Cu3), 21 mg/kg (Cu4) and 27 mg/kg (Cu5), respectively. The measured copper contents in the five experimental diets were 10.82 mg/kg, 12.63 mg/kg, 14.35 mg/kg, 15.58 mg/kg and 17.40 mg/kg, respectively. The CM was purchased from Shanghai Macklin Biochemical Co., Ltd. (Shanghai, China), with a purity of 98%. The feed production process comprised grinding, sieving, weighing, mixing, and pelletizing. Briefly, raw materials were ground to pass through a 0.25 mm sieve according to the formulation, after which they were mixed with water and oil at the prescribed proportions. The resulting mixture was then pelleted into 1 mm-diameter pellets using an SJPS56×2 pelletizer (Jiangsu Muyang Group Co., Ltd., Yangzhou, China). The pellets were air-dried under ambient conditions in a dark, cool location and subsequently stored at approximately −20 °C until used in the feeding experiment.

### 2.2. Experimental Fish and Husbandry

**Figure 1:**
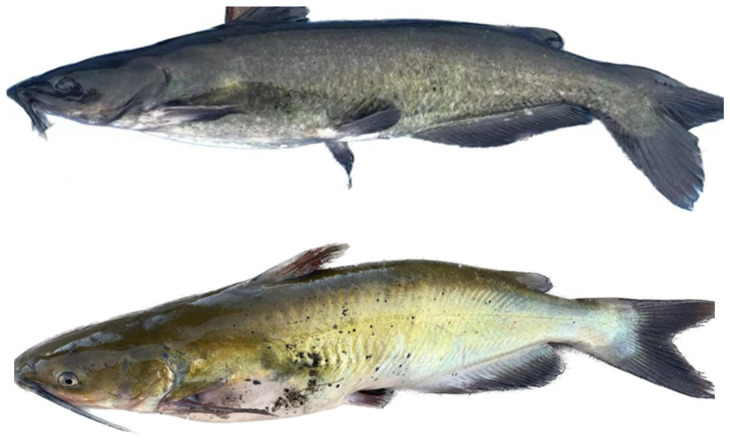
Normal channel catfish (**above**) and yellowed channel catfish (**below**).

First, channel catfish were subjected to a one-month intensive yellowing treatment in the pond (Figure 1) (by feeding them feed supplemented with exogenous MDA) [16]. The yellowed channel catfish were then screened, and their skin yellowness values were analyzed using a colorimeter (Model CR-400) (TLYON Technology Co., Ltd., Chengdu, China). Healthy channel catfish of uniform size and with essentially consistent yellowness values were selected as experimental fish. A two-week acclimatization period on the control diet was provided to help them adjust to the rearing conditions. The rearing experiment was conducted in pond cages (2 m × 2 m × 1 m) at the Nanquan Experimental Base of the Freshwater Fisheries Research Centre, Chinese Academy of Fishery Sciences. A total of 180 channel catfish with an initial body weight of 830 ± 5 g were selected and randomly assigned to five groups, each comprising three replicates of 20 individuals, totaling 15 cages, for an eight-week rearing experiment. During the experiment, five different experimental feeds were administered. All fish were size-matched at stocking to minimize inter-individual growth variation, as growth performance was not an endpoint of this pigmentation-focused study. Equal amounts were fed daily (at 07:00 and 17:00); the initial feeding rate was set at 3% of body weight and adjusted to weather conditions. Throughout the experiment, fish activity and health were observed daily. Water quality parameters in the ponds were monitored regularly; environmental parameters maintained throughout the entire rearing cycle were dissolved oxygen (>6 mg/L), water temperature (24–29 °C), ammonia nitrogen (≤0.02 mg/L) and a natural photoperiod.

### 2.3. Sample Collection and Colorimetric Analysis

At the conclusion of the 8-week rearing experiment, feeding was suspended for 24 h. First, five fish were drawn at random from every experimental cage; three served for tissue and plasma sampling, while the other two were used to determine whole-body composition. Prior to sampling, the three fish from each cage were initially anesthetized with MS-222 at a concentration of 200 mg/L, after which a color difference meter (CR-400 model) (TLYON Technology Co., Ltd., Chengdu, China) was used to measure the color values (b*) of the skin and muscle at the same location on the fish’s back, following the experimental methods described in our previous study [17]. Subsequently, blood samples were obtained from the caudal vein of the tested fish into anticoagulant tubes, and plasma was obtained via centrifugation conducted at 3000 rpm and 4 °C for a duration of 10 min, after which it was stored. Finally, skin and muscle tissue were collected from the dorsal region. Upon completion of sampling, each sample was placed in an ultra-low temperature freezer maintained at −80 °C for future experimental use.

### 2.4. Experimental Determination Method

#### 2.4.1. Determination of Nutritional Components

The feed used in the trial and the whole-fish samples were analyzed accordingly using the methods specified by AOAC (2003). A fully automatic Kjeldahl analyzer (Haineng K1100) (Jinan Haineng Instrument Co., Ltd., Jinan, China) was used to determine crude protein content by the Kjeldahl method, while crude lipid content was analyzed via the Soxhlet extraction method using a fully automatic lipid analyzer (Haineng SOX606) (Jinan Haineng Instrument Co., Ltd., Jinan, China); moisture content was determined using the constant-weight method in an oven at 105 °C; Ash content was obtained by measuring the sample weight after combustion in a muffle furnace at a temperature of 550 °C. Three replicate measurements were conducted for each component assessed.

#### 2.4.2. Analysis of Plasma Transaminase Activity and Tissue Antioxidant Markers

The measurement of plasma alanine transaminase (ALT) and aspartate aminotransferase (AST) activities was carried out on a Mindray BS-400 fully automated biochemistry analyzer (Shenzhen Mindray Bio-Medical Electronics Co., Ltd., Shenzhen, China). Antioxidant indicators in skin and muscle, including total antioxidant capacity (T-AOC), total superoxide dismutase (T-SOD), glutathione peroxidase (GSH-Px), catalase (CAT) and malondialdehyde (MDA), were tested using the appropriate test kits. The specific methods employed are detailed in the respective instruction manuals.

#### 2.4.3. Enzyme-Linked Immunosorbent Assay (ELISA) Analysis of Pigment-Related Parameters in Skin and Muscle

Uranidin, melanin, and tyrosinase were measured using ELISA kits supplied by Shanghai Enzyme-Linked Biotechnology Co., Ltd. (Shanghai, China). The methods are as follows: (1) Uranidin: purified uranidin antibodies were used to coat microtitre plates to form solid-phase antibodies. Uranidin is added to the solid-phase antibody, where it competes for binding with horseradish peroxidase (HRP)-labelled uranidin antigen. Following thorough washing, the tetramethylbenzidine (TMB) substrate is added to produce a color. The intensity of the color in the sample is inversely proportional to the uranidin content. (2) Melanin: sequentially add the sample, standard and HRP-labelled detection antibody to a microplate coated with melanin antibody. Following incubation and thorough washing, the substrate TMB is added to produce a color reaction. TMB turns blue under the catalysis of peroxidase and subsequently turns yellow under the action of acid. The intensity of the color in the sample is positively correlated with the concentration of melanin in the sample. (3) Tyrosinase: first, biotin-labelled antigen was added to the antibody-coated microplate and incubated at 37 °C for 30 min to form immune complexes. Unbound biotin-labelled antigen was then thoroughly washed away using phosphate-buffered saline with Tween 20 (PBST), followed by the addition of streptavidin-HRP. The plate was incubated again at 37 °C for 30 min to facilitate the binding of streptavidin-HRP to the biotin-labelled antigen. After washing, the bound HRP catalyzes the formation of a blue color from TMB, which subsequently turns yellow under the action of acid. The analysis and calculation of all assay parameters were performed in accordance with the instructions provided with the relevant kits. Uranidin, melanin, and tyrosinase contents were determined using the method described in our previous studies [18], which has been validated in catfish tissue and shown to correlate with changes in pigmentation.

#### 2.4.4. qRT-PCR Analysis

Skin and muscle samples underwent RNA extraction with the RNAiso Plus Kit (Nanjing Vazyme Biotech Co., Ltd., Nanjing, China). Assessment of RNA concentration and integrity was performed on a NanoDrop 2000 spectrophotometer (Thermo Fisher Scientific, Wilmington, NC, USA), with the A260/280 ratio maintained within the range of 1.8–2.0 to ensure quality standards. Using the One Step qRT-PCR SYBR Green Kit (Nanjing Vazyme Biotech Co., Ltd., China), quantitative reverse transcription PCR analysis was carried out on a CFX96 Touch instrument (Bio-Rad, Hercules, CA, USA). Given the stability of the glyceraldehyde-3-phosphate dehydrogenase (GAPDH) gene in this study, it was selected as the internal control. A complete list of the primers used in this investigation can be found in Table 2. Calculation of relative gene expression was carried out using the 2^−ΔΔCt^ method [19].

### 2.5. Data Analysis

All data are presented as means ± SD. Statistical analyses were performed using SPSS version 26.0. The cage was considered the experimental unit; values from the three fish sampled per cage were averaged to obtain a single replicate (n = 3 per treatment). Data were tested for normality (Shapiro–Wilk test) and homogeneity of variances (Levene’s test) prior to analysis. One-way ANOVA was used to evaluate treatment effects, followed by Tukey’s HSD post hoc test for multiple comparisons. Differences were considered statistically significant at *p* < 0.05.

## 3. Results

### 3.1. Effects of Copper Methionine on Plasma Transaminase Activity of Channel Catfish

**Figure 2:**
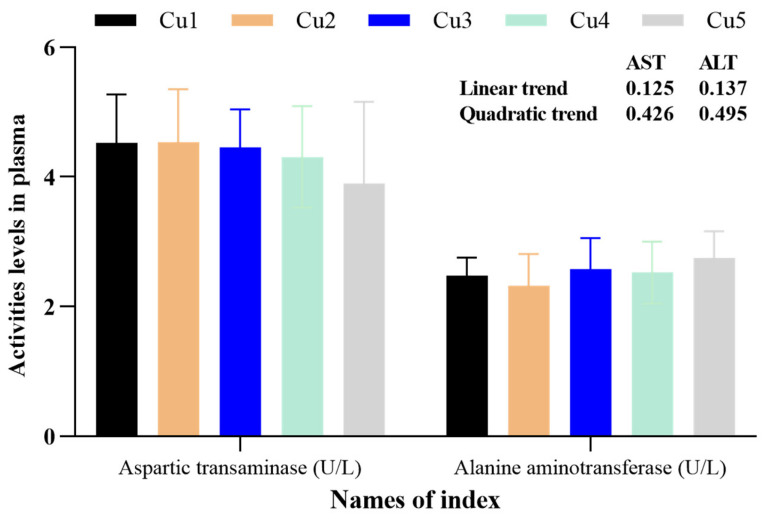
The levels of plasma AST and ALT.

Figure 2 displays the plasma biochemical results for channel catfish. Neither ALT nor AST levels differed significantly between any of the groups (*p* > 0.05).

### 3.2. Effects of Copper Methionine on Antioxidant Capacity Indexes of Channel Catfish

**Figure 3:**
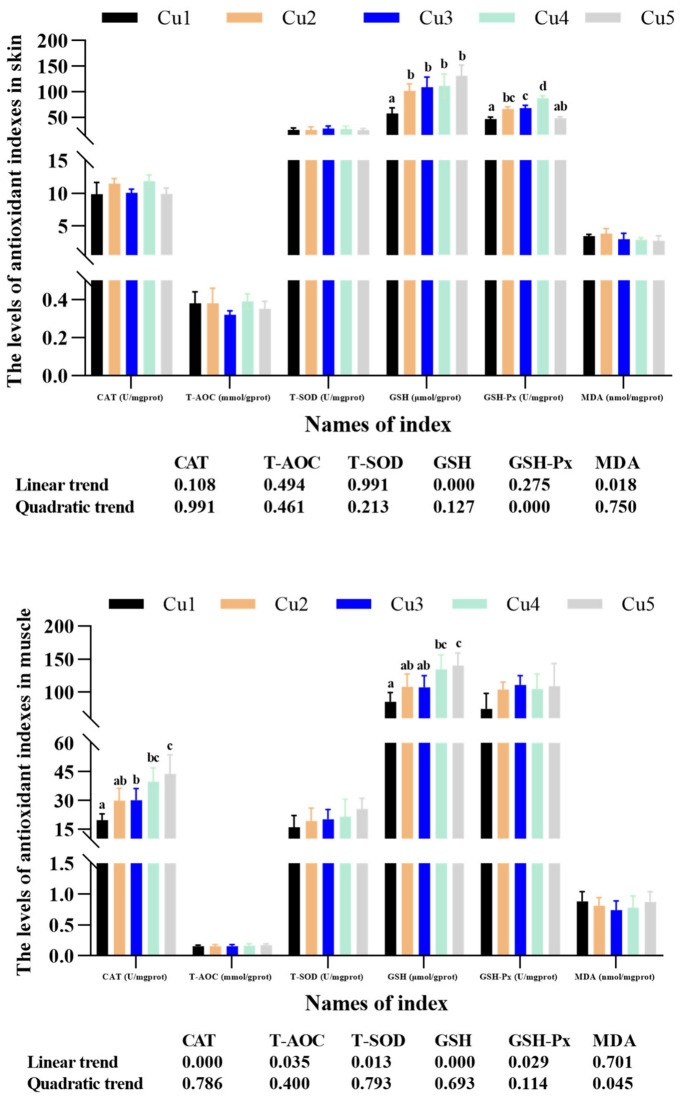
The levels of antioxidant capacity indices of skin and muscle. Different superscripted letters indicated a significant difference (*p* < 0.05).

The results of antioxidant-related parameter measurements in the skin and muscle of channel catfish are shown in Figure 3. In skin tissue, compared with the control (Cu1), the Cu2–Cu5 groups exhibited significantly higher GSH levels, which increased linearly with increasing CM addition (*p* < 0.05), and the Cu2–Cu4 groups showed significantly elevated GSH-Px activities, which increased parabolically with increasing CM addition (*p* < 0.05). In contrast, T-AOC, T-SOD, CAT, and MDA levels did not differ significantly between any groups (*p* > 0.05).

In muscle tissue, when compared to the control group (Cu1), CAT activities in the Cu3–Cu5 groups and GSH levels in the Cu4–Cu5 groups were significantly increased (*p* < 0.05), and CAT activities and GSH levels increased linearly with increasing CM addition (*p* < 0.05). However, no significant intergroup differences were found for T-AOC, T-SOD, GSH-Px, or MDA levels (*p* > 0.05).

### 3.3. Effects of Copper Methionine on Antioxidant and Immune-Related Genes in the Skin and Muscle of Channel Catfish

**Figure 4:**
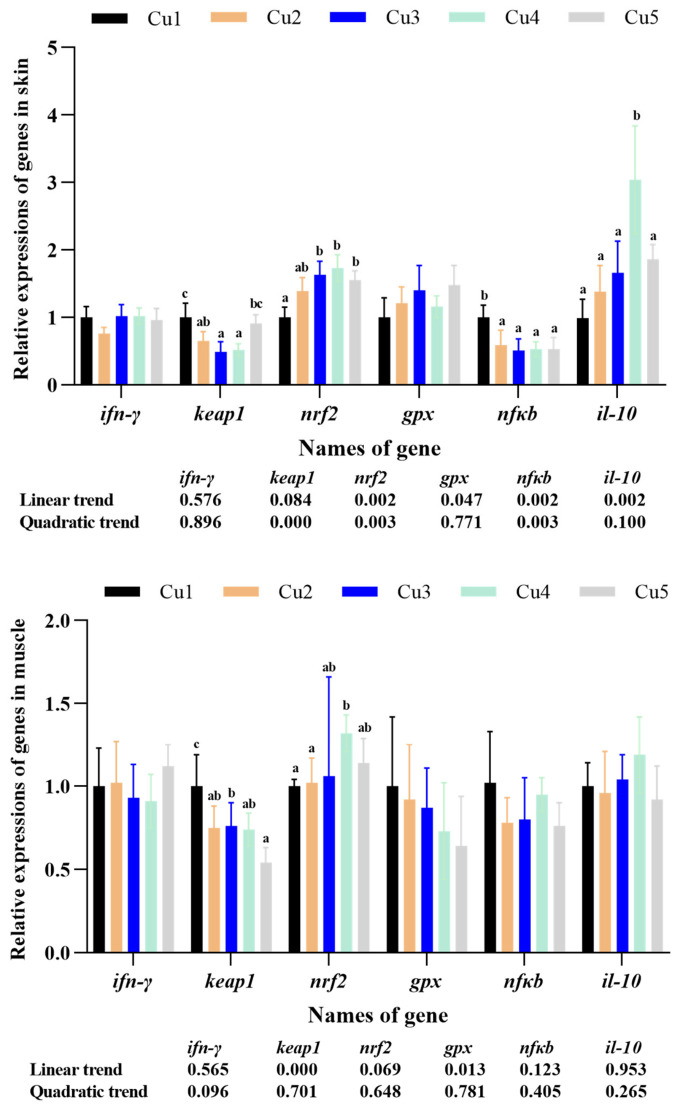
Expression of genes related to antioxidant and immunity in skin and muscle. Different superscripted letters indicated a significant difference (*p* < 0.05).

The results of the relative expression of genes associated with antioxidant capacity in skin and muscle are shown in Figure 4. In skin tissue, when compared to the control (Cu1), significant reductions were observed in the relative mRNA levels of *keap1* (Cu2–Cu4) and *nf-κb* (Cu2–Cu5) (*p* < 0.05), while significant increases were observed in *nrf2* (Cu3–Cu5) and *il-10* (Cu4) (*p* < 0.05). The expression levels of *ifn-γ* and *gpx* did not differ significantly across groups (*p* > 0.05). Furthermore, the expression levels of *keap1* and *il-10* showed a parabolic decrease and increase with increasing CM addition, respectively (*p* < 0.05), and the expression levels of *nrf2* increased in a linear or parabolic pattern, and *nfκb* decreased in a linear or parabolic pattern, respectively (*p* < 0.05).

In muscle tissue, when compared to the control (Cu1), *keap1* mRNA levels were significantly decreased in Cu3–Cu5, which decreased parabolically with increasing CM addition (*p* < 0.05), and *nrf2* mRNA levels were significantly increased in Cu4 (*p* < 0.05). The expression levels of *ifn-γ*, *gpx*, *nf-κb*, and *il-10* did not differ significantly across any of the groups (*p* > 0.05).

### 3.4. Effects of Copper Methionine on Skin and Muscle Color and Pigment Deposition in Channel Catfish

**Figure 5:**
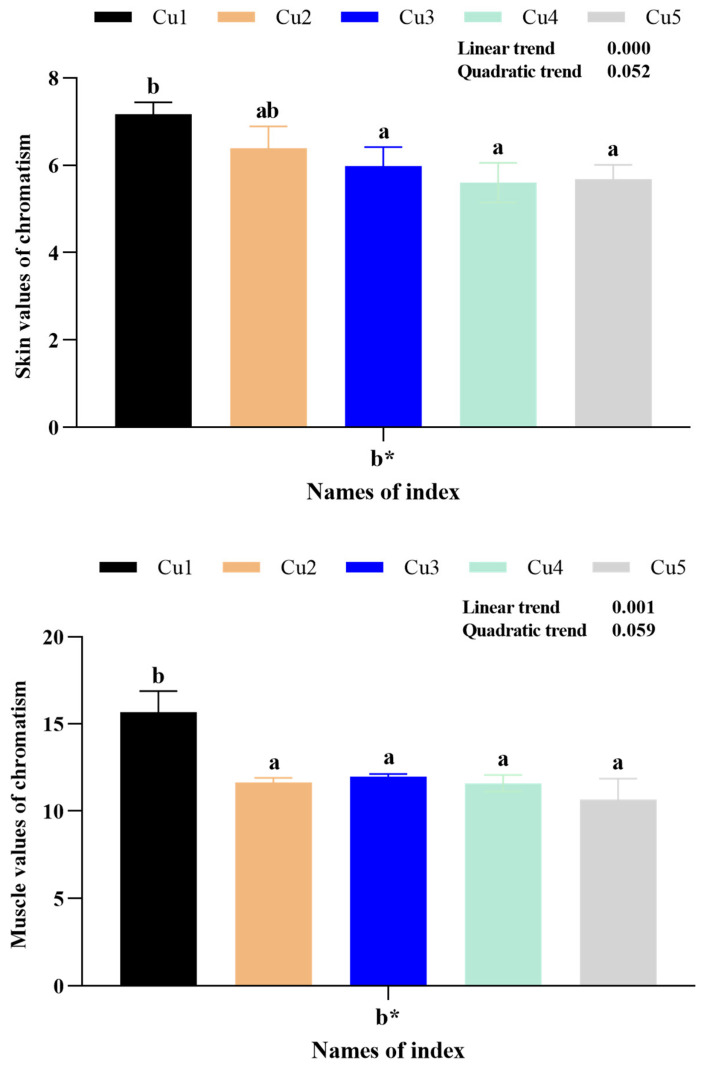
The chromaticity values in skin and muscle. b*: yellowness value. Different superscripted letters indicated a significant difference (*p* < 0.05).

The results of the colorimetric measurements of the skin and muscle of channel catfish are shown in Figure 5. In skin tissue, the Cu3–Cu5 groups showed a significant reduction in b* compared with the control group (Cu1) (*p* < 0.05). In muscle tissue, the Cu2–Cu5 groups showed a significant reduction in b* compared with the control group (Cu1) (*p* < 0.05). Furthermore, the b* value decreased linearly with increasing CM addition in both skin and muscle (*p* < 0.05).

**Figure 6:**
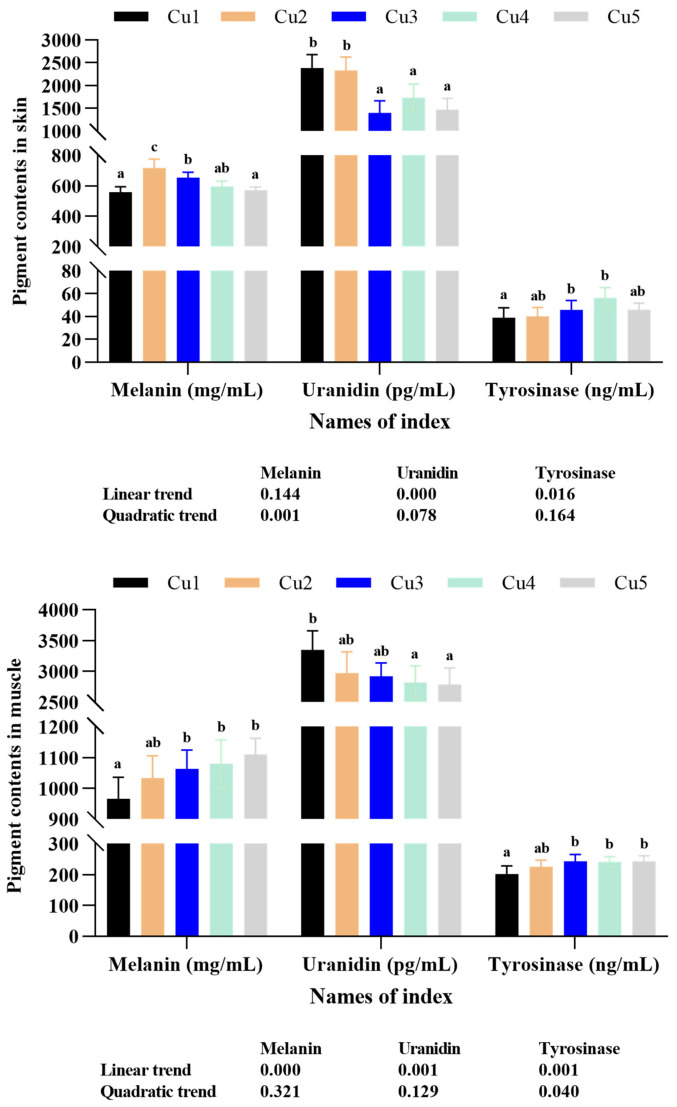
The pigmentation and tyrosinase content in skin and muscle. Different superscripted letters indicated a significant difference (*p* < 0.05).

As shown in Figure 6, skin melanin content was significantly elevated in Cu2 and Cu3 relative to Cu1, which increased parabolically with increasing CM addition (*p* < 0.05). Skin uranidin was significantly decreased in Cu3–Cu5, which decreased linearly with increasing CM addition (*p* < 0.05). Skin tyrosinase was significantly increased in Cu3 and Cu4, which increased linearly with increasing CM addition (*p* < 0.05). In muscle tissue, Cu3–Cu5 exhibited significantly higher tyrosinase and melanin levels (*p* < 0.05), whereas Cu4 and Cu5 showed significantly lower uranidin levels (*p* < 0.05). Furthermore, melanin levels increased, whilst uranidin levels decreased linearly (*p* < 0.05), and tyrosinase levels increased in a linear or parabolic pattern with increasing CM addition (*p* < 0.05).

### 3.5. Effects of Copper Methionine on Pigment-Related Genes in the Skin and Muscle of Channel Catfish

**Figure 7:**
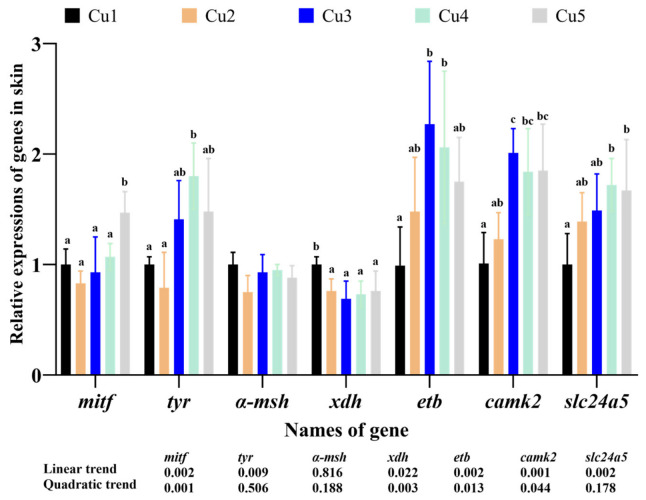

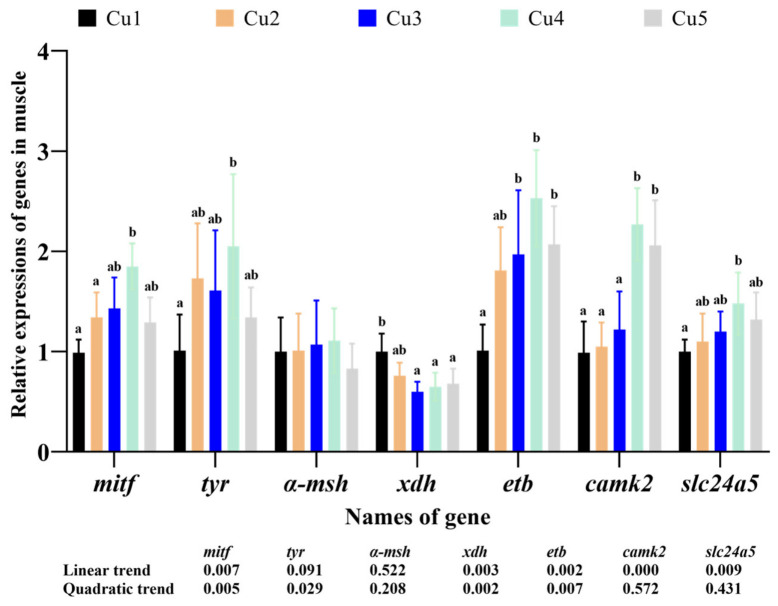
Expression of genes related to pigment synthesis in skin and muscle. Different superscripted letters indicated a significant difference (*p* < 0.05).

The results of gene expression analysis for pigment-related genes in skin and muscle are shown in Figure 7. In skin tissue, compared with the control group (Cu1), the relative expression of *mitf* mRNA was significantly increased in Cu5, *tyr* mRNA in Cu4, *etb* mRNA in both Cu3 and Cu4, *camk2* mRNA in Cu3–Cu5, and *slc24a5* mRNA in Cu4 and Cu5 (*p* < 0.05). The expression of *xdh* mRNA was significantly reduced in Cu2–Cu5 (*p* < 0.05). No significant differences were observed in *α-msh* mRNA expression among any groups (*p* > 0.05). Furthermore, *tyr* and *slc24a5* mRNA increased linearly with increasing CM addition (*p* < 0.05). In addition, *mitf*, *etb*, and *camk2* mRNA increased in a linear or parabolic pattern (*p* < 0.05), and *xdh* mRNA decreased in a linear or parabolic pattern with increasing CM addition (*p* < 0.05).

In muscle tissue, when compared to the control (Cu1), the relative mRNA levels of *mitf*, *tyr*, and *slc24a5* in the Cu4 group, *etb* in the Cu3–Cu5 groups, and camk2 in the Cu4 and Cu5 groups were all significantly elevated (*p* < 0.05). The relative expression of *xdh* mRNA was significantly decreased in the Cu3–Cu5 groups (*p* < 0.05). There were no significant differences in *α-msh* mRNA expression across any of the groups (*p* > 0.05). Furthermore, *tyr* mRNA increased parabolically and *camk2* and *slc24a5* mRNA increased linearly with increasing CM addition (*p* < 0.05). In addition, *mitf* and *etb* mRNA increased in a linear or parabolic pattern (*p* < 0.05), and *xdh* mRNA decreased in a linear or parabolic pattern with increasing CM addition (*p* < 0.05).

## 4. Discussion

Oxidative stress is one of the key factors leading to tissue damage, pigment metabolism disorders and even yellowing in aquatic animals [20,21]. As a key cofactor of Cu, Zn-SOD, copper acts as the first line of defense in the scavenging of superoxide anion radicals [2]. Adequate copper levels not only reduce radical damage to pigment cells and maintain body color stability, but are also an indispensable element in the construction of the organism’s antioxidant defense system. CAT, GSH and GSH-Px together constitute the enzymatic and non-enzymatic components at the core of the endogenous antioxidant defense system in fish. These three components work in concert to maintain the organism’s antioxidant capacity [22]. The findings from this study suggest that the addition of an appropriate amount of CM to the diet significantly increased GSH levels in the skin and muscle, as well as CAT activity in the muscle; GSH-Px activity in the skin also rose significantly. This suggests that the supplementation of exogenous CM substantially enriched the body’s reserves of non-enzymatic antioxidants and enhanced the activity of key antioxidant enzymes. MDA is the end product of lipid peroxidation and serves as a classic biomarker of oxidative stress and the extent of oxidative damage to cell membranes; elevated levels indicate that the body has sustained oxidative damage [23]. In this study, across the groups, MDA levels showed a slight downward trend in response to escalating CM content in the diet, but no significant differences were observed between them. The results demonstrate that adding 7–27 mg/kg CM to the diet not only avoided inducing oxidative stress in the skin and muscle of channel catfish but also helped maintain cell membrane integrity through elevated antioxidant enzyme activities and non-enzymatic antioxidant levels. This enhancing effect on antioxidant capacity has also been corroborated in other studies, where the addition of 4.56 mg/kg of organic copper to the diet significantly improved the antioxidant activity and physiological health of grass carp (*Ctenopharyngodon idella*), whilst promoting their growth and development [24].

The enhancement of the body’s antioxidant defense is primarily attributable to the activation of the Keap1/Nrf2/ARE signaling pathway [25]. The findings of this study demonstrate that dietary CM supplementation led to a marked downregulation of *keap1* expression in both the skin and muscle tissues of channel catfish, whilst the expression levels of *nrf2* were significantly increased. This indicates that CM could effectively promote the dissociation of Nrf2 from Keap1, thereby blocking Keap1-mediated ubiquitination and degradation of Nrf2, and consequently enhancing the body’s antioxidant defense capacity. Studies on grass carp have also corroborated this conclusion [24].

Furthermore, this study demonstrated that the relative expression of the pro-inflammatory factor *nf-κb* in the skin of channel catfish in the Cu2–Cu4 groups was significantly lower than that in the Cu1 group, whereas a marked increase in *il-10* (an anti-inflammatory factor) expression was observed in the Cu4 group. This indicates that CM, whilst enhancing antioxidant capacity, effectively suppresses inflammatory responses in the fish, thereby helping to provide a microenvironment free from inflammatory stress that is conducive to the survival and development of pigment cells and the normal synthesis of pigments. Similar findings have been reported in studies on yellow catfish (*Pelteobagrus fulvidraco*); accordingly, dietary CM was shown to significantly boost the expression of anti-inflammatory mediators and inhibit pro-inflammatory mediators in the fish, resulting in a markedly enhanced anti-inflammatory response [26].

This study reveals that as the amount of CM in the feed rises, the b* values of channel catfish skin and muscle decrease substantially. This change in color value suggests that CM could effectively alleviate yellowing in fish. Apparent changes in fish body color are essentially the result of a redistribution of various pigments within the tissues [6]. As a key cofactor of tyrosinase, copper is directly involved in and catalyzes the biosynthesis of melanin. When copper supply is insufficient, tyrosinase activity is inhibited, leading to impaired melanin synthesis and, consequently, abnormal body coloration [27]. This study demonstrated that supplementation with CM at an optimal dose substantially enhanced melanin accumulation and reduced uranidin deposition in both skin and muscle. However, cross-reactivity with other skin carotenoids was not re-assessed in the present trial, and no mass spectrometric confirmation was performed. This represents a methodological limitation; future studies employing HPLC-MS/MS would provide more definitive identification. Notably, the Cu3 and Cu4 groups exhibited a significant rise in tyrosinase content in both skin and muscle, which closely correlated with the trend in increased melanin content. This indicates that the addition of CM in the diet promotes melanin production by enhancing tyrosinase activity. This has also been confirmed by studies in fish species including Mandarin fish (*Siniperca chuatsi*) [7], zebrafish (*Danio rerio*) [28] and common carp (*Cyprinus carpio*) [29]; the spatiotemporal expression patterns of tyrosinase were found to correlate closely with changes in skin melanin intensity, with activity in dark skin regions being significantly higher than in light-colored regions. This further indicates that the expression and activity of tyrosinase are the key rate-limiting steps determining melanin deposition and the formation of body patterns in fish. Consequently, by supplementing the diet with an appropriate amount of CM, channel catfish could obtain sufficient enzyme cofactors to reactivate the inhibited melanin synthesis pathway, leading to the extensive synthesis and deposition of melanin. This also inhibits the synthesis of uranidin to a certain extent, ultimately resulting in a decrease in b* and an improvement in body color at the macroscopic level. We speculate that copper may reduce uranidin accumulation, possibly through supporting tyrosinase activity under oxidative stress, although this pathway requires direct experimental verification in future studies.

Copper regulates melanin synthesis at multiple levels. On one hand, copper serves as an essential cofactor for tyrosinase, directly binding to the enzyme’s active site to maintain its catalytic activity in melanin production. On the other hand, copper can modulate the expression of pigmentation-related genes such as *tyr* at the transcriptional level, thereby influencing the synthesis of tyrosinase protein and ultimately affecting melanin deposition in skin tissues [30]. The results of this study indicate that, in skin and muscle tissues, an appropriate level of dietary CM supplementation substantially enhanced the relative mRNA expression of *mitf* and *tyr*. MITF is widely recognized as a key regulatory factor in melanocyte development, differentiation and melanin synthesis, capable of specifically binding to the promoter region of the TYR gene to activate its transcription [31]. Studies have shown that the *mitf* and *tyr* genes play key roles in melanocyte development and melanin synthesis in the bodies of Poyang Lake loach (*Misgurnus anguillicaudatus*) [32] and the spotted sawfish (*Amphiprion ocellaris*) [33], respectively. In this study, the concurrent upregulation of *mitf* and *tyr* in the skin and muscle of the channel catfish reveals that CM does not merely participate passively in the reaction as a cofactor, but may also indirectly regulate *mitf* and *tyr* by influencing the homeostasis and transport of copper ions, thereby actively activating the entire melanin biosynthesis pathway at the transcriptional level [34]. In addition, *slc24a5* also participates in the regulation of ion balance during melanosome maturation, serving as one of the key transporters in the melanin synthesis pathway, and exhibits synergistic effects with *mitf* and *tyr* [35,36]. The *slc24a5* mRNA expression exhibits the same trend as *mitf* and *tyr*, which further confirms the above speculation. Furthermore, the findings from the preceding section indicate that the yellowing of the body color in channel catfish is accompanied by downregulation of *etb* and *camk2* gene expression, revealing a close association between these two genes and changes in body color. This study revealed that following the inclusion of a suitable dose of CM, the expression levels of both *etb* and *camk2* were significantly elevated. The Etb receptor signaling pathway is involved in melanocyte differentiation, proliferation and migration, as well as melanin synthesis in many vertebrates [37], whilst camk2, as a key hub in intracellular calcium ion signal transduction, may participate in body color regulation via the Ca^2+^/CaMKII signaling axis [20]. This suggests that CM not only promotes melanin production but may also restore the body color of yellowed channel catfish by promoting melanocyte development and distribution. In stark contrast to the upregulation of genes associated with melanin synthesis, the expression of the *xdh* gene was significantly downregulated, and *xdh* expression is closely associated with uranidin synthesis [38]. As fish body color is the combined manifestation of various pigment cells, the downregulation of *xdh* explains, at the molecular level, the significant reduction in uranidin content and the significant decrease in b* observed in the group supplemented with an appropriate level of CM. Therefore, this dual regulatory pattern—comprising enhanced melanin production coupled with inhibited uranidin synthesis—constitutes the core molecular mechanism by which methionine copper efficiently restores the body color of yellowed channel catfish.

However, excessive copper buildup in the body has the potential to cause cellular harm and negatively influence both the development and health status of aquatic species [39]. Therefore, when evaluating the effect of CM on improving body color, attention must also be paid to its impact on overall metabolism and tissue health. Blood biochemical parameters serve as important indicators of fish physiological function, nutritional metabolism and the health status of internal organs. ALT and AST are primarily found in liver cells; when the liver is damaged or diseased, these two enzymes are released into the bloodstream in large quantities [40]. In the present study, plasma ALT and AST levels in channel catfish showed no statistically significant differences across any of the experimental groups after CM supplementation, suggesting that the addition of 7–27 mg/kg of CM to the diet did not cause any apparent substantial damage or toxicity to the livers of channel catfish.

The optimal dosage range identified in this study (14–21 mg/kg CM, equivalent to 14.35–15.58 mg/kg actual copper content) offers a practical reference for feed formulation. Importantly, this range is within the legal safety limit for copper in fish feed, facilitating direct application in commercial production. However, it should be noted that while the dietary copper levels in all experimental diets were confirmed to comply with the Chinese national safety standard for fish feed (≤25 mg/kg, Ministry of Agriculture Announcement No. 2625), tissue copper accumulation—particularly in the liver and muscle—was not measured in this study. Dietary copper content alone does not fully reflect the potential for physiological accumulation over time. Therefore, the safety of methionine–copper supplementation at the tested levels, while supported by compliance with feed regulatory limits, requires direct verification through tissue residue analysis in future studies. Furthermore, given the marked price reduction and market rejection currently associated with yellowed catfish, adoption of this supplementation strategy could substantially improve farm profitability by restoring normal pigmentation and product acceptability. Future large-scale field trials under commercial conditions are warranted to validate these findings and assess economic returns.

## 5. Conclusions

In summary, copper methionine plays a significant role in regulating fish body color. By activating the Keap1/Nrf2/ARE pathway, it enhances the antioxidant and anti-inflammatory capacity of channel catfish, promotes melanin synthesis, inhibits uranidin deposition, and alleviates yellowing in channel catfish. In particular, the addition of 14 mg/kg–21 mg/kg of CM to the feed (with an actual copper content of 14.35 mg/kg–15.58 mg/kg) yielded the most favorable regulatory effects.

## Figures and Tables

**Table 1:** Ingredient and nutrient composition of experimental diets (% dry basis).

Ingredients	Additional Level (%)				
	Cu1	Cu2	Cu3	Cu4	Cu5
Fish meal ^a^	12.0	12.0	12.0	12.0	12.0
Pork powder ^a^	6.0	6.0	6.0	6.0	6.0
Soybean meal ^a^	30.0	30.0	30.0	30.0	30.0
Rapeseed meal ^a^	14.0	14.0	14.0	14.0	14.0
Wheat meal ^a^	15.5	15.5	15.5	15.5	15.5
Rice bran ^a^	15.5	15.5	15.5	15.5	15.5
Fish oil	3.5	3.5	3.5	3.5	3.5
Monocalcium phosphate	2.0	2.0	2.0	2.0	2.0
Vitamin and mineral premix ^b^	1.0	1.0	1.0	1.0	1.0
L-lysine (70%)	0.3	0.3	0.3	0.3	0.3
Choline	0.2	0.2	0.2	0.2	0.2
Methionine copper (mg/kg)	0.0	7.0	14.0	21.0	27.0
Analyzed proximate composition					
Crude protein (%)	35.8	35.7	35.8	35.9	35.8
Crude lipid (%)	8.4	8.5	8.3	8.3	8.4

Note: ^a^ Fish meal: crude protein 66.5%, crude lipid 7.8%; pork meal: crude protein 67.3%, crude lipid 12.1%; soybean meal: crude protein 45.7%, crude lipid 1.2%; rapeseed meal: crude protein 36.8%, crude lipid 2.1%; wheat flour: crude protein 13.2%, crude lipid 1.7%; rice bran: crude protein 13.5%, crude lipid 16.5%. All ingredients were purchased from Wuxi Tongwei Feed Co., Ltd. (Wuxi, China). ^b^ Vitamin and mineral premix, including vitamin premix and mineral premix. Both were purchased from Wuxi HANOVE Animal Health Products Co., Ltd. (Wuxi, China).

**Table 2:** Primer sequences for qRT-PCR analysis.

Genes	Forward (5′–3′)	Reverse (5′–3′)	Primer Source
*mitf*	CCTGGACCATGTGGCAAGTT	TGAACGTGTGTACAGGTCGG	XM_017479247.3
*tyr*	GCAGTGAGACAAACAGAGAACG	GCATGATGTTACGACGCACC	XM_053677474.1
*α-msh*	TCAACCCTCTGGCCGAAATC	GAAGGTAACCAGGCACACGA	XM_015373818.1
*xdh*	CTCGCCACAACAACATGCAA	GCTCTGACCAGGAGGGACTA	XM_017475915.1
*etb*	CAGAGCAGTCTGGGTCAAGG	ATGAGTACGTTTGGCCCGTT	XM_017474261.1
*camk2*	AATCCAGCTCCACCGTTCAG	TGATCTCCTGTTTGCGTGCT	XM_017489404.1
*ifn-γ*	GGTTTGTAATCGAGCGGGGA	TCCAAAGGTCACGCTGTACC	NM_001200178.1
*keap1*	AGAGGTACGACCCGGAAAGA	GCCGTCATAGCCACCCATTA	XM_017482240.3
*nrf2*	GGCGTGGCAAGAACAAGGTAG	TGAAGGGAGTAGTCGTTAGGG	XM_017470076.3
*gpx*	TTTGTTTGTGCCGTGTTGAGT	TGGGTGTAATCCCTGGTGGTC	NM_001200741.1
*nfκb*	TGGCGCATCCTTTGCTTAGA	AGACACAGCGGTGCATACAA	XM_017490807.1
*il-10*	TGCAGGCTTACGAAAGGGTT	CATGTCCAGCTCTCCCATGG	XM_017450800.3
*slc24a5*	ACCGCTGTGATCGCCATTAT	AAGCCCCATCACTGTGTCAG	XM_053678219.1
*gapdh*	ACCAATGAGAAGGCCTCTGC	CCTGGTGTTCTGTGTACCCC	NM_001201199.1

## Data Availability

The original contributions presented in this study are included in the article. Further inquiries can be directed to the corresponding authors.

## Abbreviations

Cu/Zn-SOD, copper-zinc superoxide dismutase; CM, copper methionine; T-AOC, total antioxidant capacity; T-SOD, total superoxide dismutase; GSH-Px, glutathione peroxidase; CAT, catalase; MDA, malondialdehyde; ALT, alanine transaminase; AST, aspartate aminotransferase; ELISA, enzyme-linked immunosorbent assay.
